# Apoptotic and Non-Apoptotic Modalities of Thymoquinone-Induced Lymphoma Cell Death: Highlight of the Role of Cytosolic Calcium and Necroptosis

**DOI:** 10.3390/cancers13143579

**Published:** 2021-07-16

**Authors:** Mimoune Berehab, Redouane Rouas, Haidar Akl, Hugues Duvillier, Fabrice Journe, Hussein Fayyad-Kazan, Ghanem Ghanem, Dominique Bron, Philippe Lewalle, Makram Merimi

**Affiliations:** 1 Laboratory of Experimental Hematology, Institut Jules Bordet-Université libre de Bruxelles, 1000 Brussels, Belgium; redouane.rouas@bordet.be (R.R.); hugues.duvillier@bordet.be (H.D.); hussein.fayyad-kazan@bordet.be (H.F.-K.); dominique.bron@bordet.be (D.B.); philippe.lewalle@bordet.be (P.L.); makram.merimi@bordet.be (M.M.); 2 Department of Biology, Functional Biology, Katholieke Universiteit Leuven, 3000 Leuven, Belgium; 3 Department of Cellular and Molecular Medicine, Katholieke Universiteit Leuven, 3000 Leuven, Belgium; Haidar.Akl@kuleuven.be; 4 Department of Biology, Lebanese University, Hadath 40016, Lebanon; 5 Laboratory of Oncology and Experimental Surgery, Institut Jules Bordet-Université libre de Bruxelles, 1000 Brussels, Belgium; fabrice.journe@bordet.be (F.J.); ghanem.ghanem@bordet.be (G.G.); 6 Genetics and Immune Cell Therapy Unit, LBBES Laboratory, Faculty of Sciences, University Mohammed Premier, Oujda 60000, Morocco

**Keywords:** thymoquinone, diffuse large B cell lymphoma (DLBCL), apoptosis, ER-stress, non-apoptotic cell death, necroptosis

## Abstract

**Simple Summary:**

Diffuse large B cell lymphoma (DLBCL) represents the most common type of non-Hodgkin lymphoma with a high curability rate. However, 40% of patients will relapse or exhibit refractory disease, and compromised apoptotic pathways is among the prognosis-worsening factors. Therefore, drugging non-apoptotic modalities might be therapeutically promising. Thymoquinone (TQ) has been reported to promote apoptosis in cancer cells. Herein, we show that TQ selectively kills DLBCL cells, either cell lines or primary lymphoma cells bearing resistance features to standard treatment. Investigations show that, although TQ induced apoptotic markers, non-apoptotic death was the major mechanism responsible for TQ-induced cellular demise. We demonstrate critical and selective roles of cytosolic calcium and necroptosis in TQ-induced non-apoptotic cell death. Finally, TQ exhibits an improved selectivity profile over conventional chemotherapy. Collectively, this work provides new insights into the mode of action of TQ and points to the therapeutic relevance of non-apoptotic modalities as a fail-safe mechanism for pro-apoptotic DLBCL therapies.

**Abstract:**

Targeting non-apoptotic modalities might be therapeutically promising in diffuse large B cell lymphoma (DLBCL) patients with compromised apoptotic pathways. Thymoquinone (TQ) has been reported to promote apoptosis in cancer cells, but little is known about its effect on non-apoptotic pathways. This work investigates TQ selectivity against DLBCL cell lines and the cell death mechanisms. TQ reduces cell viability and kills cell lines with minimal toxicity on normal hematological cells. Mechanistically, TQ promotes the mitochondrial caspase pathway and increases genotoxicity. However, insensitivity of most cell lines to caspase inhibition by z-VAD-fmk (benzyloxycarbonyl-Val-Ala-Asp-fluoromethyl ketone) pointed to a critical role of non-apoptotic signaling. In cells dying through non-apoptotic death, TQ increases endoplasmic reticulum (ER) stress markers and substantially increases cytosolic calcium ([Ca^2+^]_c_) through ER calcium depletion and activation of store-operated calcium entry (SOCE). Chelation of [Ca^2+^]_c_, but not SOCE inhibitors, reduces TQ-induced non-apoptotic cell death, highlighting the critical role of calcium in a non-apoptotic effect of TQ. Investigations showed that TQ-induced [Ca^2+^]_c_ signaling is primarily initiated by necroptosis upstream to SOCE, and inhibition necroptosis by necrostatin-1 alone or with z-VAD-fmk blocks the cell death. Finally, TQ exhibits an improved selectivity profile over standard chemotherapy agents, suggesting a therapeutic relevance of the pro-necroptotic effect of TQ as a fail-safe mechanism for DLBCL therapies targeting apoptosis.

## 1. Introduction

Diffuse large B cell lymphoma (DLBCL) is the most common form of non-Hodgkin’s lymphoma (NHL) and accounts for 30 to 40% of adult NHL cases [1]. Gene expression profiling classifies DLBCL into at least three subtypes: activated B cell-like (ABC), germinal center B-cell like (GCB), and primary mediastinal lymphoma (PMBL), and each has distinct oncogenic mechanisms. A combination chemotherapy regimen comprising cyclophosphamide, doxorubicin, vincristine, and prednisone (CHOP) in conjunction with rituximab remains the first-line treatment for ABC and GBC DLBCL [2]. The five-year overall survival is 40%, 60–70%, and over 60% for the ABC, GBC, and PMBL subtypes, respectively [3]. Despite receiving aggressive therapy, many patients are not cured, and their outcome is generally poor. Because chemotherapy-induced cell death primarily involves apoptotic pathways, disrupting these pathways can largely interfere with treatment efficacy [4,5]. Bypassing apoptosis resistance remains a critical challenge for improving the response rate of refractory and relapsed DLBCL [4]. Therefore, strategies targeting non-apoptotic modalities could either synergize with conventional therapies or compensate for their failure.

Thymoquinone (TQ) is the most abundant active compound from the medicinal plant Nigella sativa and has demonstrated promising anticancer potential against various malignancies in vitro and in vivo [6,7]. The anticancer activity of TQ occurs through different molecular mechanisms, primarily via cell growth inhibition and cell death induction. The most well-characterized cell death pathways associated with TQ involve activation of the intrinsic apoptosis pathway with deregulations of BCL-2/BCL-XL and BAX ratios [8,9,10,11,12,13,14]. At present, in contrast to the pro-apoptotic effect of TQ, the role of non-apoptotic cell death is poorly characterized, and underlying mechanisms are unclear. TQ-promoted non-apoptotic cell death has been reported in glioblastoma, colon cancer, and prostate cancer. Lysosomal cell death in glioblastoma and autophagic cell death in squamous carcinoma have been speculated as the primary mechanisms of TQ [15,16,17,18]. The present study reports evidence concerning the selective cytotoxicity of TQ in DLBCL bearing resistance features to conventional chemotherapy through concomitant induction of apoptotic and non-apoptotic modalities. The non-apoptotic modality shown here appeared predominant and occurred remarkably in TQ-sensitive cells, whereas fewer sensitive cells typically died by apoptosis. Our investigations evidenced the role of necroptotic mechanisms in the non-apoptotic cell death induced by TQ, which constitutes a novel insight into the molecular mechanism of TQ cytotoxicity.

## 2. Materials and Methods

### 2.1. Cell Lines and Culture Conditions

The human GCB-DLBCL cell lines Toledo, SUDHL4, and HT were generously provided by Dr. Haidar Akl (Laboratory of Molecular and Cellular Signaling, Department of Cellular and Molecular Medicine, KU Leuven, Leuven, Belgium), and WSU-NHL cells (ACC 58) were purchased from DSMZ cell bank (Braunschweig, Germany). Peripheral blood mononuclear cells (PBMCs) were purified from blood collected from healthy volunteers and separated on a Ficoll gradient using Histopaque-1077 from Sigma-Aldrich (Diegem, Belgium). The volunteers provided informed consent, and the study protocol was approved by the ethics committee of the hospital. All cell lines were grown in complete culture medium containing RPMI 1640 culture medium supplemented with 10% heat-inactivated fetal bovine serum, 2 mM L-glutamine, and 50 units/mL penicillin/streptomycin. Cell lines were incubated at 37 °C with 100% humidity and 5% CO_2_ and were routinely checked for Mycoplasma sp contamination (Mycoplasma Detection Kit-QuickTest, Biotol).

### 2.2. Primary Lymphoid Cells from Patients

Primary cells from a relapsed DLBCL patient were isolated from peripheral blood of the patient. Primary lymphoid cells from cHL (classical Hodgkin’s Lymphoma), NSHL (nodular sclerosing Hodgkin’s lymphoma), and MZLs (marginal zone lymphomas) were isolated from lymph node biopsies for diagnosis using a gentleMACS™ Dissociator system (Miltenyi Biotec). Isolated cells were plated in complete culture medium, and lymphoid cells were identified with anti-CD45 and anti-CD-19 antibodies during FACS analysis.

### 2.3. Reagents

Thymoquinone (TQ), EGTA, 4-PBA, Pepstatin-A, and Calpeptin were from Sigma-Aldrich. BAPTA-AM, Fluo4-AM, and Pluronic F-127 (20% solution in DMSO) were purchased from Thermo Fisher (Gent, Belgium). ALLM was from TOCRIS-biosciences. Rhod2-AM, BTP2, ML9, 3MA, and antibody against TNFα were from Abcam; z-VAD-fmk was purchased from BD-Pharmingen; necrostatin-1 (Nec-1) and Thapsigargin (TG) were from Cayman (Ann Arbor, MI, USA); SP000125 was from MedChemExpress; and RU360 was from Calbiochem. 4-Hydroperoxy Cyclophosphamide (4-HC) was from Santa Cruz Biotechnology-Bio-Connect (Huissen, The Nederland), and doxorubicin was provided by Jules Bordet Institute Hospital.

### 2.4. Cell Viability Assay

Cell viability was determined using MTS per the manufacturer’s directions (CellTiter 96^®^ Aqueous One Solution, Promega, Leiden, Belgium). The cells were seeded at 30 × 103 cells per well in 96-well plates, treated, and incubated for 48 h. Then, 20 µL of MTS was added per well, and the plates were incubated again for 3 h. The absorbance was measured with a microplate photometer reader (Thermo Fisher Scientific, Erembodegem, Belgium) at a wavelength of 490 nm. Cell viability was measured by comparing the absorbance values of drug-treated cells to those of vehicle-treated controls (DMSO) set at 100%. The IC_50_ values were calculated from sigmoidal dose response curves using Prism 6.0 (GraphPad, San Diego, CA, USA).

### 2.5. Cell Death Assay

Cells were seeded into 12-well plates at 0.8 × 106 cells per mL per well. Treated cells were harvested and washed twice with PBS-EDTA. Cell death was determined using annexin-V-FITC and PI (propidium iodide) staining according to the manufacturer’s guidelines (BD Pharmingen, Erembodegem, Belgium). To determine cell death in B lymphocytes, PBMCs were immunostained with anti-CD45 (e-Fluor 780) and anti-CD19 (APC) antibodies (eBioscience, San Diego, CA, USA) prior to annexin-V-FITC/PI staining. Stained cells were analyzed using flow cytometry (NAVIOS-Beckman Coulter, Indianapolis, Indiana), and the generated data were analyzed using KALUZA software (Beckman Coulter).

### 2.6. Analysis of DNA Damage, Apoptosis, and Cell Cycle Distribution

Detection of apoptosis, DNA damage, and cell cycle distribution was achieved following guidelines from Apoptosis, DNA Damage, and Cell Proliferation Kit (BD Pharmingen). In brief, cells were incubated with 10 µL of BrdU (1 mM) per 1 mL of cell culture for 40 min, after which the cell suspension was washed twice with PBS and staining buffer (BD Pharmingen). Collected cells were subjected to three steps of fixation and permeabilization. Then, fixed and permeabilized cells were treated with DNAse for 1 h at 37 °C; washed; and labeled with anti-BrdU, anti-γH2AX (pSer139), and anti-cleaved-PARP (c-PARP) antibodies for 30 min at room temperature. The cells were then washed and resuspended in the stain buffer (FBS) containing DAPI prior to flow cytometry analysis.

### 2.7. RNA Isolation and qPCR Analysis

Total cellular RNA was isolated from 4 × 106 cells using Tripure isolation reagent per the manufacturer’s protocol (Roche Diagnostics GmbH, Mannheim, Germany). RNA was cleaned, purified, and treated with DNAse using a kit from Zymo Research (Irvine, CA, USA). The RNA concentrations were measured using a NanoDropTM 1000 spectrophotometer (Thermo Scientific, Waltham, MA, USA), and the integrity and quality of the RNA were evaluated using capillary electrophoresis. To identify dysregulated genes, quantitative RT-PCR arrays (Human Apoptosis pathway 96 StellARray™ qPCR Array) from Harbor Bioscientific-Lonza (Verviers, Belgium) were used (the list of genes is shown in the Supplementary Table). Briefly, RNA was reverse transcribed to cDNA using M-MLV reverse transcriptase in qScriptTM cDNA SuperMix (QuantaBio- VWR International bvba, Leuven, Belgium) following the manufacturer’s instructions. Then, 500 ng of cDNA was mixed with SYBR-green reagent (Thermo Scientific), and 20 µL of the resulting mixture was added to a 96-well microarray plate. qPCR was performed in a StepOnePlus™ Real-Time PCR System (Applied Biosystems Inc., CA, USA) with the following program: 1 cycle of a holding stage at 50 °C for 2 min and 95 °C for 5 min and 40 cycles of the amplification stage at 95 °C for 15 s and 60 °C for 1 min. Comparative analysis was then performed between untreated and treated conditions using the online Global Pattern Recognition™ (GPR) Data Analysis Tool.

To detect spliced XBP1 mRNA, PCR was conducted using Immolase DNA polymerase (Bioline, London, UK) following the manufacturer’s instructions. β-Actin was used as a loading control. The primers used in these experiments were as follows: human Xbp-1, 5′-GGGTCCAAGTTGTCCAGAATGC-3′ (forward) and 5’-TTACGAGAGAAAACTCATGGC-3’ (reverse); and β-actin, 5′- GCTCGTCGTCGACAACGGCTC-3′ (forward) and 5′- CAAACACTCATCTGGGTCATCTTCTC-3′ (reverse). The PCR cycle was as follows: 1 cycle at 95 °C for 5 min; 36 cycles of 95 °C for 1 min, 58 °C for 30 s, and 72 °C for 30 s; and a final extension at 72 °C for 5 min. The PCR products were resolved on either a 2% agarose/1× TAE gel or a commercialized gel (E-Gel EX 2% Agarose SYBR Gold II, Thermo Fisher Scientific).

### 2.8. Protein Extraction and Immunoblotting

Total protein was extracted from 10 × 106 cells using M-PER mammalian extraction reagent supplemented with phosphatase/protease inhibitor cocktails (Thermo Fisher Scientific; Waltham, MA, USA), and the protein concentration was determined using a BCA Protein Assay (Thermo Fisher. Western blotting (WB) was performed as previously described [19]. In brief, equal amounts of cell lysate protein were separated on an SDS-polyacrylamide gel and transferred to a nitrocellulose membrane. Next, immunoblotting was performed: first with primary antibodies under gentle shaking overnight and then with the appropriate secondary antibody (HRP-coupled) for 2 h at room temperature. The immunoblotting results were visualized with a chemiluminescence detection system (LAS-3000 FUJIFILM). Primary antibodies against the following proteins were used: caspase-3, caspase-9, BIP/GRP78, and AIF (Cell Signaling Technology Europe-Bioke; Leiden, Nederland); BCL-2, β-actin, and α-tubulin (Santa Cruz Biotechnology-Bio-Connect); HPSPA1A (Abnova; Taipei, Taiwan); and HMGB1 (ABCAM; Cambridge, UK). Secondary antibodies (HRP-coupled) against rabbit-IgG and mousse-IgG were used (GE Healthcare).

### 2.9. Subcellular Fractionation

Briefly, 3–2.5 × 106 cells were subjected to cell fractionation following the protocol of Cell Fractionation kit (ABCAM). Cytoplasmic, mitochondrial, and nuclear fractions were processed and subjected to WB using Apotrack antibody cocktail (ABCAM) containing antibodies targeting cytochrome c, the cytosolic marker GAPDH, and the mitochondrial markers CVα and PDHE-α.

### 2.10. Cytosolic Calcium Analysis

Cells (0.8 × 106/mL) were plated in 12-well plates; after culturing for a time, they were harvested and washed twice with PBS. The cells were then loaded with 5 µM Fluo-4 AM (F-127, 20% solution in DMSO) in loading buffer (HBSS supplemented with 5% FBS) at 37 °C for 1 h, washed twice with PBS, resuspended in HBSS buffer, and incubated at room temperature for an additional hour, followed by analysis via flow cytometry.

### 2.11. Statistical Analysis

Analysis of variance (ANOVA) and Student’s *t*-test were used where applicable to evaluate the significance of differences between data. Analysis was performed with GraphPad Prism software.

## 3. Results

### 3.1. Cytotoxicity of TQ toward Cancer and Normal Cells

**Figure 1:**
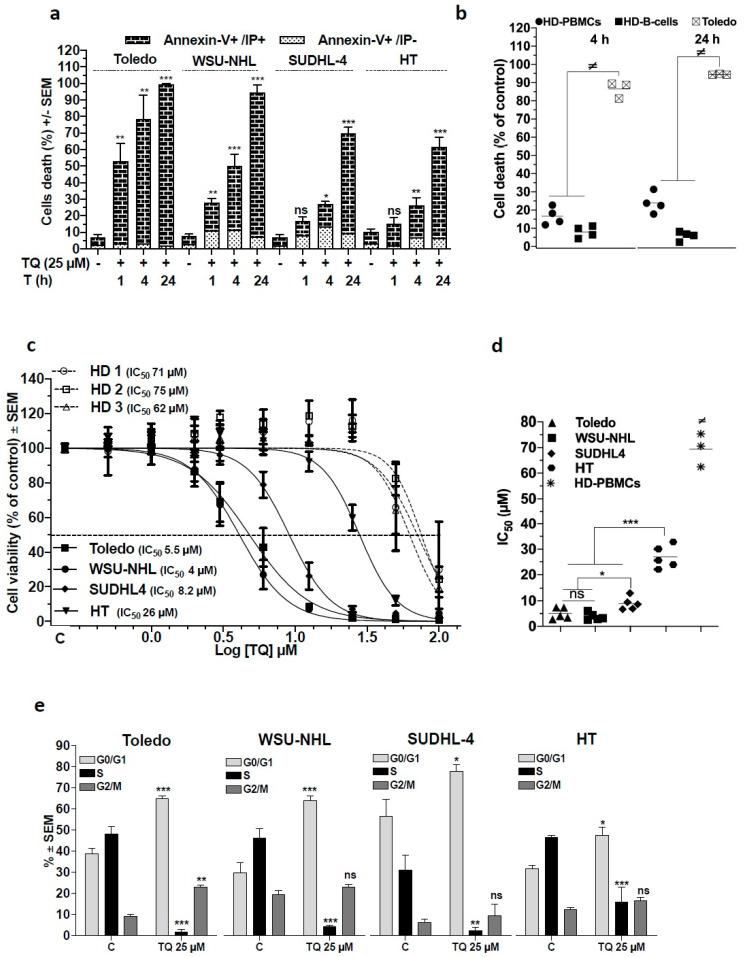
Effect of thymoquinone (TQ) on cell death and cell proliferation of GCB-DLBCL cell lines Toledo, WSU-NHL, SUDHL-4, and HT, and healthy hematological cells. (**a**,**b**) Cell death analysis: Cells were incubated in the presence of the drug vehicle (DMSO) or thymoquinone (TQ), then cell death was analyzed via flow cytometry after Annexin-V-FTIC/PI staining. Dead cells were identified as Annexin-V-FITC- and PI-positive cells. (**a**) Data are the mean of at least three independent experiments for DLBCL cells lines treated with 25 µM TQ for 1, 4, and 24 h. (**b**) Cell death of TQ in normal cells compared with Toledo cells: Peripheral blood mononuclear cells (PBMCs) were isolated from four healthy donors (HD), and activated using 5 µg of PHA and 20 units of IL-2/mL for 40 h before treatment. Cells were then treated with the vehicle or with 25 µM TQ for 4 and 24 h, and analyzed for cell death; B cells were identified with antibodies against CD45 and CD19. (**c**) The cell viability was monitored using MTS assays after incubation with increasing concentrations of TQ for 48 h (0.5 to 100 µM). The dose–response curves for cell viability were established and IC_50_ values were calculated using GraphPad Prism 6 software (cell lines: *n* = 5, PBMCs: 3 healthy donors). (**d**) Comparison of IC_5__0_ values of TQ between DLBCL cell lines and the cell lines versus activated PBMCs. (**e**) Effect of TQ on cell cycle: Cells were incubated with BrdU for 40 min and stained with anti-BrdU antibody and DAPI to identify the distribution of cell cycle phases; cells were analyzed via flow cytometry (*n* = 3). Student’s *t*-test: ns *p* > 0.05, * *p* < 0.05, ** *p* < 0.01, *** *p* < 0.001; ANOVA: ≠ *p* < 0.05.

We investigated the effect of TQ in a panel of established human GCB-DLBCL cell lines: Toledo, WSU-NHL, SUDHL-4, and HT. These cells express a complex profile of the BCL-2-family proteins, which have been recognized to play a decisive role in cell susceptibility to apoptotic stimuli [20]. Initially, we assessed the cell-killing kinetic of 25 µM TQ using annexin-V-FITC and PI staining followed by flow cytometry and found that TQ efficiently induced cell death in a time-dependent manner in the different cell lines. In Toledo and WSU-NHL cells, cell death occurred early, beginning at 1 h and reaching up to 40% at 4 h and more than 90% at 24 h post-treatment. In SUDHL-4 and HT cells, significant cell death (25% of cells) occurred at 4 h and peaked at 24 h post-treatment (over 60% of cells) (Figure 1a). Early apoptosis (annexin-V-FITC+/PI−) was minimally induced, in contrast with the late apoptosis and necrosis (V-FITC+/PI+) that appeared prevalent in TQ-induced cell death. We then assessed TQ-induced cell death in activated healthy donor (HD) PBMCs and found that 25 µM TQ generated low cell death rates in HD cells either whole PBCMs or B cells compared with malignant B cells (Toledo) where the cell mortality rate was significantly greater (Figure 1b), indicating a selective potency against DLBCL cells.

To clarify the extent of cell susceptibility to TQ and TQ selectivity, we analyzed the viability of cell lines and proliferating HD-PBMCs using MTS assays. The 48 h IC_50_ values in DLBCL cells lines ranged from 4 to 26 µM and approximately 70 µM in HD-PBMCs, indicating a marked level of selectivity (Figure 1c,d). TQ appeared more potent against WSU-NHL, Toledo, and SUDHL-4 cells, with IC_50_ values of 4, 5.5, and 8.2 µM, respectively, while against HT cells, TQ was least potent, with an IC_50_ of 26 µM. In accordance with these data, the cell death EC_50_ revealed sensitivity of Toledo, WSU-NHL, and SUDHL-4 cells (EC_50_ 5.4, 7.7, and 11 µM, respectively) and lower sensitivity of HT cells (EC_50_ 24 µM) (Figure S1a,b). TQ cytotoxicity was further characterized by cell cycle analysis, which showed that TQ provoked the strongest decrease in cells at S phase concurrent with arrest at G0/G1 in all cells (Figure 1e). Together, these results demonstrate that TQ cytotoxicity was preferentially directed toward DLBCL cells and involved cell death induction and cell viability decrease concomitant to growth arrest. Remarkably, the cell lines presented differential cell susceptibility, indicating a gradual activation of the underlying cytotoxic mechanism.

### 3.2. TQ Promotes Expression of Markers of Mitochondrial Apoptosis and Genotoxicity

**Figure 2:**
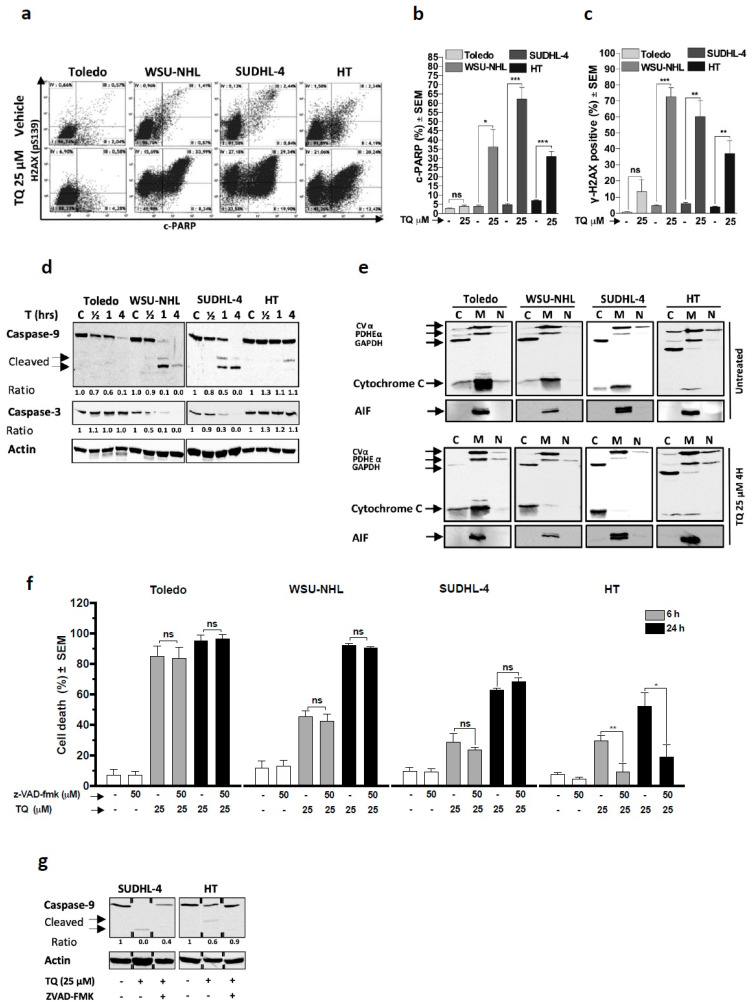
Role of the apoptotic pathway in the TQ-induced cell death effect: (**a**–**c**) Effect of TQ on apoptosis and DNA damage: Cell lines were treated with 25 µM TQ for 4 h and then immunostained with antibodies targeting cleaved-PARP and phophorylated-H2AX (γ-H2AX) to detect apoptosis and DNA damage, respectively. (**a**) A c-PARP/γ-H2AX dot plot profile for DLBCL cells treated with the vehicle or TQ; (**b**,**c**) the average of data from at least three independent experiments for c-PARP and γ-H2AX, respectively. (**d**) Effect of TQ on the caspase cascade: GCB-DLBCL cell lines were treated with 25 µM for 1/2 h, 1 h, and 4 h, then cell lysate proteins were subjected to WB using anti-caspase-9 antibody and anti-caspase-3 antibody. Actin was used as the loading control. (**e**) Effect of TQ on translocation of mitochondrial proteins: cytosolic, mitochondrial, and nuclear fractionations were performed on untreated cells and cells treated with 25 µM TQ for 4 h. Cell extracts were then subjected to WB using anti-AIF antibody and ApoTrack antibody cocktail containing antibodies against GAPDH (cytosolic marker), CVα and PDHE-α (mitochondrial markers), and cytochrome c. (**f**) Effect of the pan-caspase inhibitor z-VAD-fmk on TQ-induced cell death: DLBCL cell lines were pretreated with 50 µM z-VAD-fmk for 1 h and then treated with 25 µM TQ alone or in combination with z-VAD-fmk for 6 and 24 h. Cell death was monitored by Annexin-V/PI staining and flow cytometry analysis (*n* = 3). (**g**) Effect of z-VAD-fmk on TQ-induced caspase-9 activation: HT and SUDHL-4 cells were pretreated with 50 µM z-VAD-fmk or vehicle for 1 h and then treated with 25 µM TQ for 4 h. Cell lysates from the different conditions were then subjected to WB analysis to detect caspase-9 and actin. Student’s *t*-test: ns *p* > 0.05, * *p* < 0.05, ** *p* < 0.01, *** *p* < 0.001; all experiments were repeated at least three times.

Apoptosis has been widely recognized as a critical death mechanism for TQ-induced cytotoxicity, notably through the mitochondrial pathway [8,9,13,14,18,21,22,23,24,25,26]. We first analyzed apoptosis (c-PARP) and genotoxicity (pH2AX) and found that 4 h of TQ treatment resulted in a significant increase in both processes in almost cell lines except the Toledo line, which did not manifest a meaningful increase (Figure 2a–c). In accordance with these findings, WB analysis revealed activation of caspases subsequent to cytochrome c release in WSU-NHL, SUDHL-4, and HT cells upon TQ treatment. TQ concomitantly increased the activated caspase-9 level and decreased the pro-caspase-3 and -9 levels in parallel with cytochrome c release, which occurred early and prominently in WSU-NHL and SUDHL-4 cells, but manifested later and less impressively in HT cells (Figure 2d–e). In Toledo cells, cytochrome c release was unremarkable upon TQ treatment, and subsequent caspase activation was not detected. These results indicate the ability of TQ to induce mitochondrial apoptosis parallel with genotoxicity and suggest the participation of a caspase-independent cell death (CID) mechanism, at least in Toledo cells.

Because Bcl-2 is a major antagonist of mitochondrial apoptosis, we asked whether this can influence the cytotoxic effect of TQ. WB analysis revealed that TQ did not remarkably affect Bcl-2-expression at time points when the cells died and its expression did not fit with cell susceptibility because it was not detectable in the least sensitive HT cells (Figure S2a). Next, the cell death effect of TQ against HT and HT-Bcl2 cells (HT cells stably transfected with the Bcl-2 gene) was monitored, and the results showed that both lines were similarly sensitive (Figure S2b,c). These results indicate that Bcl-2 is minimally involved in the susceptibility of cells to TQ and further support a substantial role of non-apoptotic pathways in TQ-induced cell death.

### 3.3. Non-Apoptotic Cell Death Is Key in TQ-Induced Cell Death in Sensitive Cells

To verify the involvement of CID, we examined the effect of TQ on AIF release from the mitochondria. Cells treated with TQ did not exhibit altered mitochondrial localization of AIF (Figure 2e), which eliminated this connection to the observed CID. Next, we questioned whether apoptosis critically contributed to the cell-killing effect of TQ. Experiments showed that caspase inhibition with z-VAD-fmk failed to block TQ-mediated cell death in Toledo cells at 6 h and 24 h and in WSU-NHL and SUDHL-4 cells, notwithstanding the detectable apoptotic markers in these cells (Figure 2f). In contrast, z-VAD-fmk significantly rescued cell death at 6 h and 24 h in HT cells, indicating a critical role of apoptosis in this cell line. The efficiency of caspase inhibition by z-VAD-fmk was investigated via WB, and the results showed that z-VAD-fmk efficiently prevented TQ-induced caspase-9 activation in SUDHL-4 and HT cells (Figure 2g) Overall, these results suggest that the critical role of activated caspases after TQ treatment was restricted to HT cells and strongly indicate a potential role of non-apoptotic cell death in the sensitive cell lines.

### 3.4. Molecular Patterns Associated with the Cell Response to TQ

**Figure 3:**
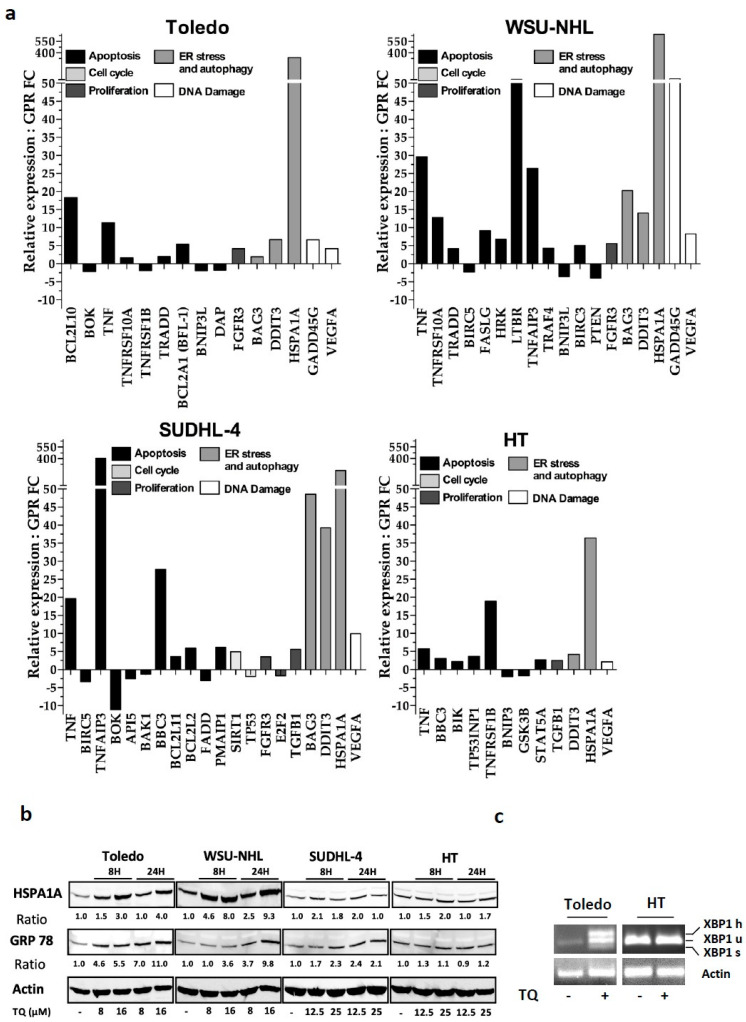
Molecular pattern associated with cell line responsiveness to TQ. (**a**) Effect of TQ on common genes related to proliferative and cell death pathways: cDNA-reversed RNA from cells treated with the vehicle or 25 µM TQ for 4 h were submitted to qPCR analysis using commercialized plates for gene pattern analysis. The graphs represent the significant transcriptionally up- and downregulated genes in the different cell lines. Values represent fold changes in gene expression between control and treated groups. The means of significant genes from three independent experiments are shown, with a minimal cut-off of 1.5. (**b**,**c**) Effect of TQ on ER stress. (**b**) Cells were treated with the indicated doses of TQ for 8 h and 24 h. WB was then performed using antibodies against GRP78 and HSPA1A, and actin was used as the loading control. (**c**) Detection of XBP-1 splicing via semiquantitative RT–PCR on total RNA extracted from Toledo and HT cells treated with 25 μM TQ. The represented PCR products are XBP1u (unspliced XBP1), XBP1s (spliced XBP1), and XBP1h (hybrid XBP1); actin was used as the loading control.

To investigate prominent changes in molecular pathways upon TQ exposure, we sought to identify TQ-modulated genes involved in key proliferative and cell death signaling pathways. Real-time qPCR showed significantly dysregulated genes (Figure 3a), which are classified according to their involvement in the indicated cellular processes. TQ increased the expression of pro-apoptotic genes involved in either the intrinsic or extrinsic pathways and induced an increase in anti-apoptotic genes, but to a lesser extent. TQ-modulated genes included an increase in the expression of genes associated with cell cycle arrest, proliferation inhibition, and genotoxic stress. Importantly, induced HSPA1A, DDIT3, and BAG-3 expression is associated with the unfolded protein response (UPR) [27], and TQ enhanced their expression more prominently in the sensitive cell lines. DDIT3 and HSPA1A mRNA showed an increase in HT cells, but less impressively. These findings suggest that TQ-induced proteotoxic stress is likely overshadowed by a functional ER disturbance in sensitive cells. To further strengthen this evidence, levels of the ER stress markers GRP78 and HSPA1A and mRNA splicing of XBP1 were analyzed. WB results revealed continuous elevation of GRP-78 expression upon TQ exposure in sensitive cells with less striking fluctuations in HT cells (Figure 3b). Under similar conditions, TQ treatment strongly increased HSPA1A levels in Toledo and WSU-NHL, but modestly in HT and SUDHL-4 cells. These findings indicate TQ-induced proteotoxic stress that perturbed ER functioning more prominently in sensitive cells. Consistently, qPCR analysis of XBP1 showed that TQ increased spliced-XBP1 (sXBP1) and hybrid-XBP1 (hXBP1) levels in Toledo in contrast with HT cells, in which both genes were less visible (Figure 3c). Because ER stress can contribute to cell death modalities [28,29], we investigated this eventuality. The results showed that ER stress inhibition by 4-PBA as well as JNK inhibition by SP600125 both failed to reduce TQ-induced cell death in Toledo cells (Figure S4a,b), suggesting that ER stress is inducible via an upstream mechanism.

### 3.5. Non-Apoptotic Cell Killing by TQ Relies Primarily on Cytosolic Calcium

**Figure 4:**
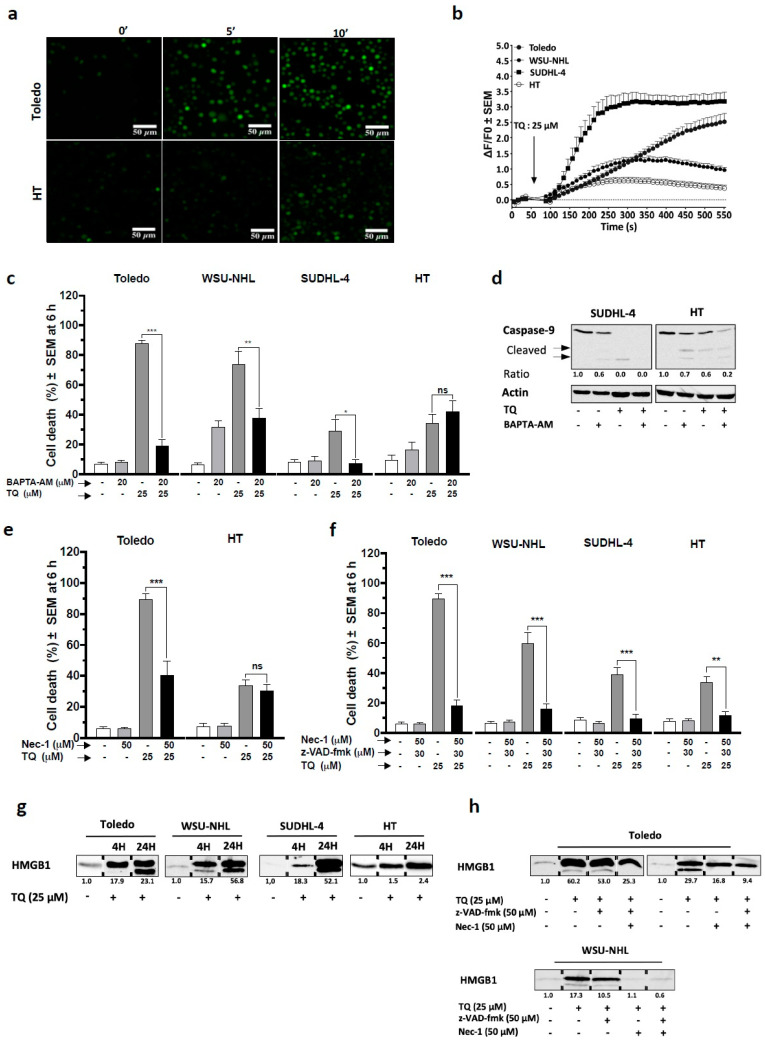
Role of cytosolic calcium efflux and necroptosis-like activity in TQ-induced non-apoptotic cell death. (**a**,**b**) Cytosolic calcium mobilization was monitored by fluorometric analysis using the Ca^2+^ indicator FLUO-4 AM. (**a**) A fluorescence imaging of Toledo and HT cells treated with 25 µM TQ for 10 and 30 min. (**b**) Cells were loaded with FLUO-4 AM, and emitted fluorescence was monitored from 1 s to 540 s via flow cytometry in all cell lines; treatment with 25 µM TQ was performed at 60 s. The data are visualized as the peak intensity of the fluorescence at 10 s intervals. The mean data from six independent experiments are shown. (**c**) Effect of the intracellular calcium chelator BAPTA-AM on TQ-induced cell death: cells were pretreated for 1 h with 20 µM BAPTA-AM or the vehicle and then treated with 25 µM TQ for 6 h; cell death was monitored as above, and data are the average of at least three independent experiments. (**d**) Effect of BAPTA-AM on TQ-induced caspase-9 activation: SUDHL-4 and HT cells were pretreated with 20 μM BAPTA-AM for 1 h and then treated with 25 μM TQ for 4 h; cell lysates were then subjected to WB analysis using antibodies against caspase-9 and actin. (**e**,**f**) Effect of nec-1 associated or not with z-VAD-fmk on TQ-induced cell death 6 h post-treatment: in (**e**), Toledo and HT cells were pretreated with nec-1 alone, and in (**f**) (*n* = 4), cell lines were pretreated with nec-1 and z-VAD-fmk (*n* = 4). (**g**,**h**) Effect of TQ on HMGB1 release: an equal number of cells were plated, and supernatants were subjected to WB analysis using anti-HMGB1 antibody: in (**g**), supernatant analysis of cells treated with TQ or the vehicle for 6 h, and in (**h**), supernatant analysis of Toledo and WSU-NHL cells pretreated or not with nec-1 and z-VAD-together or separately and then treated with TQ for 6 h. Student’s *t*-test: ns *p* > 0.05, * *p* < 0.05, ** *p* < 0.01, *** *p* < 0.001.

We asked whether variation in cytosolic calcium could be involved in TQ effects because this can impede ER homeostasis and promote cell death [30,31]. Our findings showed that Toledo cells exposed to TQ exhibited visibly enhanced Fluo-4 fluorescence at 5- and 10-min post-treatment, indicating an increase in [Ca^2+^]_c_, whereas in HT cells, the changes were less impressive (Figure 4a). Consistent with these observations, continuous cytosolic calcium imaging via flow cytometry revealed an immediate increase in [Ca^2+^]_c_ upon TQ treatment in sensitive cells, with a modest increase in HT cells (Figure 4b). To support these findings, we examined the effect of TG, a calcium-mobilizing agent (Figure S3), and the results showed that TG elicited a visible [Ca^2+^]_c_ increase in all cells, indicating successful dye loading. Next, we examined whether [Ca^2+^]_c_ could be involved in the cell killing activity of TQ using BAPTA-AM, an intracellular calcium chelator. Strikingly, the results showed that BAPTA-AM reduced TQ-induced cell death (6 h) at least by 50% in sensitive cells, but failed to rescue HT cells (Figure 4c). Remarkably, BAPTA-AM produced drastic protection from cell death in Toledo cells, which were virtually free of apoptosis, but failed to protect HT cells, which succumbed to apoptosis. Together, these data indicate a specific role of calcium in the non-apoptotic killing effect of TQ. In line with this evidence, WB analysis revealed that BAPTA-AM failed to protect both sensitive (SUDHL-4) and less sensitive (HT) cells from TQ-induced caspase-9 activation (Figure 4d).

### 3.6. Necroptosis Plays a Key Role in TQ-Induced Non-Apoptotic Cell Death

Cytosolic calcium has been reported to be critical during non-apoptotic cell death modalities, including necroptosis [32,33,34,35]. Strikingly, here, necroptosis inhibition by necrostatin-1 (nec-1) significantly slowed the cell death rate by up to half in Toledo cells, in which apoptosis criticality was clearly distinguishable compared with HT cells that appeared insensitive to the protective effect of nec-1 (Figure 4e). To avoid the compensatory effect of apoptosis, TQ cell death was examined in the presence of both nec-1 and z-VAD-fmk. The results showed that the association of nec-1 with z-VAD-fmk sharply reduced TQ-induced cell death in all cells at 6 h post-treatment (Figure 4f). These findings indicate that necroptosis plays a critical role in the non-apoptotic modality of TQ. To further improve necroptosis promotion, we first analyzed the effect of TQ on extracellular release of HMGB1 because necroptosis induces release of damage-associated molecular patterns (DAMPs), including HMGB1 [36]. We observed that TQ provoked an important release of HMGB1 in sensitive cells after 4 h and 24 h of treatment, while in HT cells, this occurred less impressively (Figure 4g). Then, we studied the effect of nec-1 and z-VAD-fmk on TQ-induced HMGB1 release in the highly sensitive cell lines Toledo and WSU-NHL. The results showed that z-VAD-fmk alone failed to reduce HMGB1 release in both cell lines; meanwhile, nec-1 alone or combined with z-VAD-fmk visibly reduced HMGB1 release from these cells (Figure 4h). These observations corroborated the necroptotic features of TQ-induced non-apoptotic cell death.

### 3.7. TQ Provoked Depletion of ER-Calcium SOCE Activation Subsequently to Necroptosis

**Figure 5:**
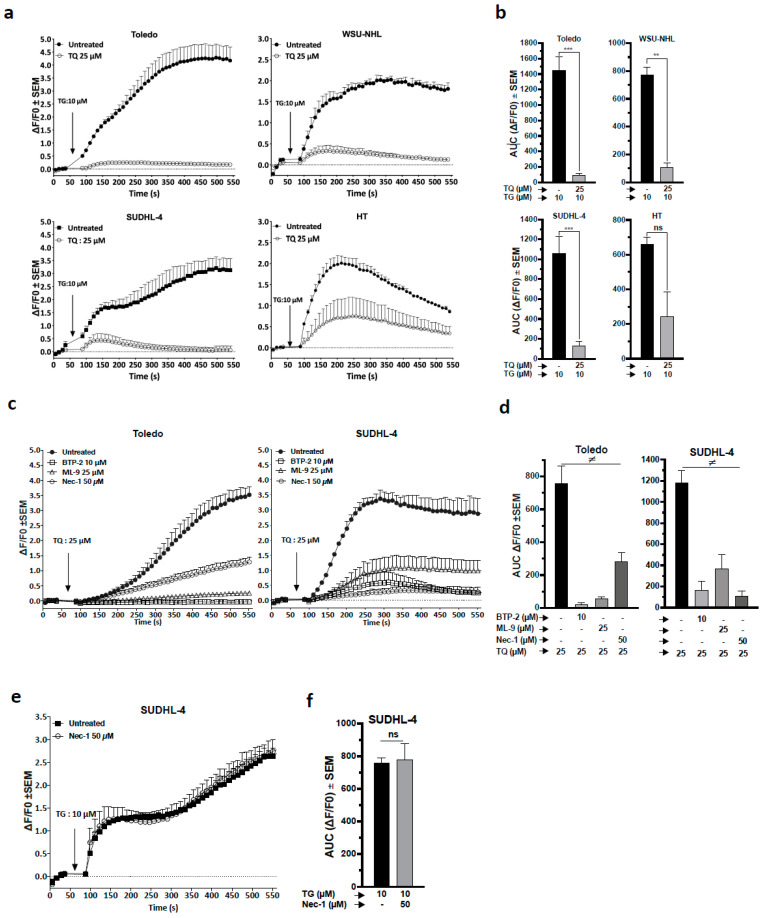
Role of ER-calcium depletion and necroptosis in TQ-mediated calcium efflux. (**a**–**f**) Cells were pretreated as indicated for 1 h, then cytosolic calcium was continuously monitored via flow cytometry in the presence of the indicated drug added at 60 s. For all experiments, the curve represents the peak intensity of the fluorescence at 10 s intervals, and the corresponding area under the curve (AUC) was calculated. (**a**) Effect of TQ on TG-induced ER calcium release in cell lines: the cells were pretreated with 25 µM TQ or DMSO for 1 h, then cytosolic calcium was analyzed in the presence of 10 µM TG. (**b**) AUC calculated from the calcium response curves in A. (**c**) Cytosolic calcium mobilization induced by TQ in Toledo and SUDHL-4 cells pretreated with SOCE inhibitors (BTP-2 and ML-9) or an RIPK1 inhibitor (nec-1), and the respective AUCs are shown in (**d**). (**e**) Cytosolic calcium mobilization induced by TG in SUDHL-4 cells pretreated with nec-1, and the corresponding AUC is shown in (**f**). Student’s *t*-test: ns *p* > 0.05, * *p* < 0.05, ** *p* < 0.01, *** *p* < 0.001; ANOVA: ≠ *p* < 0.05; all experiments were repeated at least three times.

To obtain insights into the relationship linking functional ER disturbance and [Ca^2+^]_c_ during TQ treatment, we analyzed the effect of TQ on TG-induced ER calcium release because TG could potentially induce a [Ca^2+^]_c_ increase when ER stores are not depleted. The results showed that cells exposed to TQ exhibited severely impaired TG-induced [Ca^2+^]_c_ increase in all cells and slightly less in HT cells, which was further indicated by the area under the curve (AUC) analysis (Figure 5a,b). These data indicate that TQ depleted the ER calcium pool more prominently in sensitive cells. Next, the involvement of store-operated calcium entry (SOCE) during TQ-induced calcium response was tested using BTP-10 and ML-9, specific inhibitors of ORAI-I and STIM1, respectively. We found that both ML-9 and BTP-10 strongly reduced TQ-induced [Ca^2+^]_c_ release in Toledo and SUDHL-4 cells, and the AUC analysis demonstrated a significant and severe reduction in releasable calcium (Figure 5c,d). These findings indicate that TQ provokes SOCE [Ca^2+^]_c_ and thus further validate the TQ effect on the ER calcium pool. Consequently, the contribution of SOCE to TQ-mediated cell death was examined. The results showed that SOCE inhibitors failed to minimize TQ-induced cell death (Figure S4b), which indicated that SOCE is not primarily responsible for TQ-induced cell death, but instead is mediated through an upstream mechanism.

We next examined the role of necroptosis in the TQ-induced cell calcium response. The results showed that nec-1 sharply reduced TQ-induced [Ca^2+^]_c_ increase in Toledo and SUDHL-4 cells; AUC analysis indicated a significant reduction in releasable calcium in both lines by at least two halves (Figure 5c,d). These findings highlight the subsequent role of necroptosis in the TQ-induced cell calcium response. To verify these findings, TG-releasable calcium was analyzed in the presence of nec-1 in SUDHLL-4. The results showed that nec-1 did not change TG-releasable calcium (Figure 5e,f), indicating that nec-1 did not interfere directly with the SOCE process, thus confirming the role of necroptosis as an upstream inducer of TQ-induced [Ca^2+^]_c_ increase. Collectively, these results elucidate a key role of TQ-induced necroptosis as an origin event orchestrating [Ca^2+^]_c_ increase through ER calcium depletion and SOCE activation. Importantly, the results demonstrate a closed correlation between the susceptibility of cell lines and events associated with necroptosis activation.

### 3.8. The Potential Therapeutic Benefit of TQ Cytotoxicity

**Figure 6:**
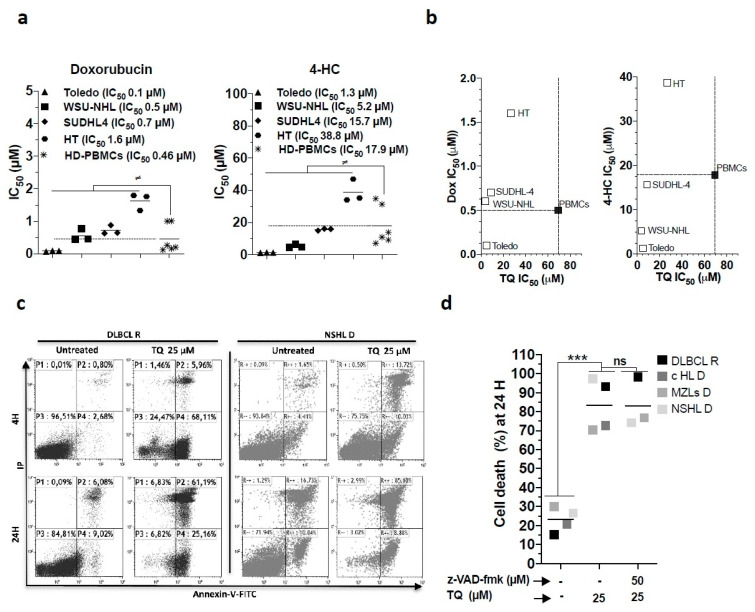
Potential benefit of the cytotoxic effect of TQ. (**a,b**) Effect of 4-hydroxy-cyclophosphamide (4-HC) and doxorubicin (Dox) on cell viability of cell lines and activated HD PBMCs. (**a**) Comparison of IC_50_ of 4-HC and Dox in cell lines and HD PBMCs. (**b**) IC_50_ plots comparing TQ versus Dox and TQ versus 4-HC. The dotted line represents the cut-off that refers to the IC_50_ inhibitor in HD PBMCs and defines the selectivity profile of each inhibitor. (**c**,**d**) Cell death effect of TQ alone or combined with z-VAD-fmk in primary lymphoid cells, including DLBCL at relapse (R), cHL (classical Hodgkin’s lymphoma), NSHL (nodular sclerosing Hodgkin lymphoma), and MZLs (marginal zone lymphomas) at diagnosis (D). (**c**) Annexin-V-FITC/IP plot profile of untreated cells and TQ-treated DLBCL R and NSHL D cells, primary lymphoid blasts were identified with antibodies against CD45 and CD19. (**d**) Cell death plot comparison of untreated cells and cells treated with 25 µM TQ or z-VAD-fmk. Student’s *t*-test: ns *p* > 0.05, * *p* < 0.05, ** *p* < 0.01, *** *p* < 0.001; ANOVA: ≠ *p* < 0.05, PBMCs (6 HD) vs. cell lines (*n* = 3).

We asked whether TQ-induced non-apoptotic cytotoxicity could potentially be relevant in the context of DLBCL therapy. We first assessed alternative sensitivity patterns to TQ by profiling cell lines and HD PBMCs sensitivity toward the routinely administered chemotherapeutic agents 4-hydroxy-cyclophosphamide (4-HC) and doxorubicin (Dox). Considering HD cells’ susceptibility, the results evidenced the ability of TQ to selectively kill lines that present potential resistance to 4-HC and Dox (Figure 6a,b). In fact, it showed that most cell lines were resistant to Dox, except the Toledo line, whereas HT and to some extent SUDHL-4 cells exhibited a resistant pattern toward 4-HC. We then assessed the effects of TQ on the death of primary lymphoid cells from patients diagnosed with lymphoid malignancies, including a patient with a second relapse of DLBCL (leukemic stage). The results showed that TQ promoted a more rapid and higher cell death rate in all primary lymphoid blasts (Figure 6c,d), indicating their sensitivity pattern. Interestingly, TQ-induced cell death is likely non-apoptotic because z-VAD-fmk failed to rescue the cells from TQ-induced cell death (Figure 6d). These observations highlight the ability of TQ to kill heterogeneous lymphoid cell populations and support the potential activity of TQ against lymphoma cells bearing a resistant phenotype.

## 4. Discussion

Successful anticancer therapies are well known to involve efficient and specific tumor cell death [37,38]. In DLBCL, disruption of the apoptotic pathway is often accompanied by therapy resistance and poor clinical outcomes. In this work, TQ co-induced apoptotic and non-apoptotic cell death in malignant GCB-DLBCL cell lines exhibiting a heterogeneous profile of apoptosis regulators and resistance features to standard chemotherapeutic agents [20,39]. We showed that TQ kills DLBCL lines and decreases their viability in a dose- and time-dependent manner concurrently with cell cycle arrest. These effects were significantly more pronounced in DLBCL cells than in normal hematological cells. Previous studies reported differential cytotoxicity of TQ between cancerous cells and normal cells, including PBMCs [8,18,22,40]. However, to the best of our knowledge, this is the first study in which the selectivity of TQ was evaluated against normal hematological cycling cells.

To date, the anticancer effects of TQ have been extensively studied in several malignancy models. TQ has been reported to modulate many key oncogenic pathways and consequently interfere with cancer cell death, proliferation, and angiogenesis [41]. The oncogenic pathways that are most frequently abrogated by TQ and thought to be responsible for its anticancer effects involve the cell transducers p53, NF-κB, PPARγ, STAT3, MAPK, and PI3K/AKT [42]. TQ has been reported to mitigate NF-κB signaling [43,44,45,46,47,48], block STAT3 signaling [11,13,14,49], and promote DNA damage-mediated activation of p53 or p53 alternative pathways [17,22,23,50,51]. The mechanism through which TQ kills cancer cells has garnered increasing attention in preclinical studies, and in nearly all of them, the cell-killing effect of TQ was related to mitochondrial apoptosis [8,9,13,14,18,21,22,23]. Likewise, we showed in our work that TQ induces apoptosis via the intrinsic pathway subsequent to cytochrome c release and intrinsic caspase cascade activation. TQ-mediated apoptosis occurred simultaneously with DNA damage in cell lines undergoing mitochondrial and caspase events. However, here, our findings were in line with evidence pointing to the critical contribution of non-apoptotic modalities in mediating TQ cytotoxicity. First, TQ promoted a potent pro-death effect in Toledo cells, which exhibited no apoptosis or genotoxicity. Second, our investigations revealed a noncrucial role of anti-apoptotic Bcl-2 and the apoptosis CID inducer AIF in TQ-induced cell death. Finally, insensitivity to the general caspase inhibitor z-VAD-fmk confirmed operational non-apoptotic mechanisms in sensitive cells. Therefore, the contribution of TQ-induced-apoptosis appeared exclusively restricted to less sensitive cells (e.g., HT cells).

TQ-induced cytotoxicity has been attributed to non-apoptotic cell death in other cancer cell lines; however, the critical triggers remain ambiguous [15,16,17,18]. Here, TQ-induced non-apoptotic cell death was pertinently associated with elevated ER stress, but did not primarily contribute to TQ cytotoxicity, thus indicating an upstream mechanism. Surprisingly, we observed a prominent increase in [Ca^2+^]_c_ upon TQ treatment that was more robust in sensitive cells, where it appeared selectively critical for non-apoptotic cell death. Hence, cytosolic calcium chelation dramatically prevented TQ-induced cell death in sensitive cells, but did not reverse caspase activity. In this respect, [Ca^2+^]_c_ influx has been shown to be critical in orchestrating many non-apoptotic modalities [32,33,34,35], particularly during necroptosis, in which it acts as an early driver. During necroptosis, the necrosome complex containing RIPK1/3 and MLKL is activated [52,53]. Then, MLKL oligomerizes in the cytoplasmic membrane and provokes membrane rupture and the release of numerous intracellular molecules, including DAMPs [54]. We here identified necroptosis as a critical mechanism for TQ-induced non-apoptotic cell death. Our results showed that nec-1-mediated RIPK1 inhibition alone or combined with z-VAD-fmk abrogated TQ-induced non-apoptotic cell death and extensively reduced the DAMP expression pattern. Whether the triggering signal responsible for necroptosis arises intrinsically or extrinsically is not quite clear and requires further investigation. Nevertheless, Al-Khatib et al. reported that DR5 knockdown failed to protect against TQ-induced cell death [8,9]. We showed that anti-TNF-α antibody failed to protect against TQ-mediated cell death (Figure S4d), although TQ-provoked TNF mRNA increases ruled out a primary role of TNFR in TQ-induced necroptosis. Rather, it has been reported that autocrine release of TNF results from upstream lethal signals mediated by necroptosis or genotoxicity [36]. Further, in the present study, z-VAD-fmk did not augment cell susceptibility to TQ and was not required to promote the observed necroptosis features, especially because caspase inhibition by z-VAD-fmk has been shown to be required for enabling necroptosis in some cancer cells, particularly during death-receptor induced necroptosis [55].

We have evidenced a relationship between TQ-induced ER dysfunction, [Ca^2+^]_c_ release, and necroptosis. Our data suggest that necroptosis is the substantial trigger responsible for TQ-induced calcium signaling including ER calcium depletion and activation of SOCE. Depletion of ER calcium activates the ER sensor, STIM1, which in turn initiates SOCE by recruiting CRAC channels to take up calcium from the extracellular calcium pool [56]. Here, TG-induced [Ca^2+^]_c_ release was highly incapacitated upon TQ treatment, primarily in sensitive cells, indicating depletion of ER stores, which could subsequently provoke ER stress. Consistently, inhibition of STIM1 and ORAI1 attenuated the TQ-induced calcium response and unsurprisingly failed to reverse the cell death, supporting the presence of an upstream inducer. Previously, many downstream signaling factors associated with ER stress and [Ca^2+^]_c_ spike have been attributed to TQ cytotoxicity. Activation of autophagy and lysosomal proteases [15,16,18], as well as the stress pathways JNK and p38, which could be related to ER stress, have been reported [15,17,43,57]. These changes might reflect underlying events disturbing ER function; however, the authors did not define the upstream UPR pathways. In our investigations, pharmacological inhibition of cathepsins and autophagy failed to prevent TQ-induced cell death (Figure S4b,c). These observations further corroborate speculations pointing to an underlying mechanism responsible for both the calcium response and the related death modality. Increased [Ca^2+^]_c_ is an early feature of necroptosis [54] and is primarily promoted by MLKL through the calcium channel TRMP7 and has even been shown to be activated downstream to RIPK1 [58]. Accordingly, RIPK1 inhibition by nec-1 selectively reduced [Ca^2+^]c induced by TQ, but not by TG. Together, these investigations highlight the prominent role of necroptosis in the TQ-induced calcium spike as well as its pro-death effect. Notably, the present study evidenced for the first time the prominent role of necroptosis in promoting SOCE and provoking functional disturbance of the ER. Supporting this evidence, Faouzi et al. demonstrated the role of TRMP7, a key player in necroptosis-mediated calcium signaling, in the SOCE process [59]. Furthermore, our data show substantial mitochondrial calcium uptake upon TQ treatment, which could quickly produce a bioenergetic crisis by enforcing mitochondrial dysfunction (Figure S5a,b).

Considering our findings, we sought to examine the therapeutic relevance of the TQ-mediated non-apoptotic and necroptotic killing effect. We found that TQ has a better selectivity profile toward DLBCL cells than Dox and 4-HC, thus indicating a potency of TQ against resistant cells. Extensively, we showed the ability of TQ to actively and rapidly kill primary cancer cells, including refractory DLBCL cells. To some extent, the non-apoptotic and pro-necroptotic effect of TQ could be beneficial against CHOP-resistant DLBCL, but this notion requires further validation with a greater number of primary samples and in vivo experiments. As a proof of concept, it is interesting to stress that a clinical trial was conducted to evaluate the benefits of Nigella sativa seeds oil, in which TQ is the major bioactive component, in reducing cardiovascular risks in hypertensive patients [60]. Nevertheless, TQ is poorly water-soluble. To overcome the challenges associated with phytochemicals’ bioavailability, and avoiding unspecific binding, different formulations of TQ nanoparticles are tested against several types of cancer, whereby the studies showed greater effectiveness of TQ nanoparticle than free TQ [61]. These formulations included nanostructured lipid carriers (NLCs), solid lipid nanocarriers (SLNs), polymeric, niosomal, and liposomal [62]. In the case of lymphomas, the conjugation of a nanoparticle with an anti CD19 or CD20 antibody could lead to a targeted delivery of thymoquinone in the lymphoma cells.

## 5. Conclusions

**Figure 7:**
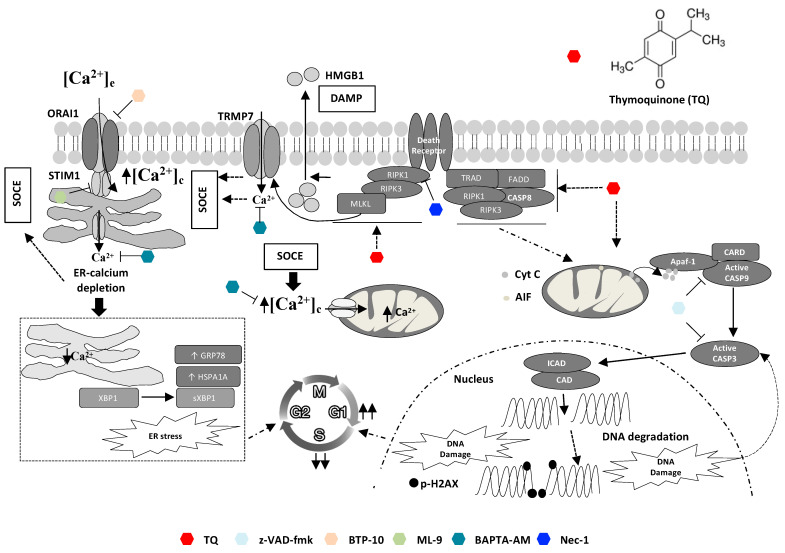
Graphical summary of involved mechanisms in TQ-induced cell death. TQ promotes both apoptotic and non-apoptotic cell death modalities: an early and critical role of necroptosis and cytosolic calcium. Non-apoptotic cell death involving necroptosis signaling is the most critical cell killing mechanism underlying TQ-cytotoxicity. Necroptotic features appear early after TQ treatment as evidenced by an early increase in cytosolic calcium and latterly by extracellular release of HMGB1. Both BAPTA-AM, a cytosolic calcium chelator, and nec-1, a RIPK1 inhibitor, reversed TQ-induced non-apoptotic cell death. Cytosolic calcium increase during TQ treatment involves activation of SOCE after ER-calcium depletion, which also leads to ER stress as manifested by increase of GRP78, HSPA1A, and sXBP1; BTP-10 and ML-9, inhibitors of STIM1 and ORAI, respectively, blocked SOCE. Promotion of SOCE occurs more likely downstream to necroptosis initiation and is potentially mediated by TRMP7; nec-1 reduced TQ-induced SOCE. Apoptotic effect of TQ occurs through the intrinsic pathway as highlighted by cytochrome c release from the mitochondria and caspase-9/3 activations leading to PARP cleavage and potentially to DNA damage after DNA fragmentation by ICAD activated in turn by active caspase-3. The pan-caspase inhibitor z-VAD-fmk blocked caspase activity, but failed to reverse the cell death in most cells except the less sensitive cells. DNA damage and ER stress and their upstream inducers cooperate to TQ-induced cell cycle arrest.

In summary, the present study demonstrates that TQ can kill DLBCL cells through more than one death mechanism (Figure 7). On one hand, TQ-induced apoptosis appeared minimally responsible for TQ cytotoxicity. On the other hand, TQ triggered non-apoptotic death mediated by the necroptotic program, requiring cytosolic calcium and ER function disturbance, appeared critical for TQ cytotoxicity. Our work suggests that TQ-mediated non-apoptotic cell death serves as a fail-safe mechanism when apoptosis routes are compromised. Therefore, pro-necroptotic therapy could constitute a promising approach to target DLBCL malignancies that are resistant to pro-apoptotic chemotherapy agents.

## Data Availability

All data are available at the Experimental Hematology Laboratory, Jules Bordet Institute, Heger Bordet street 1, 1000 Brussels Belgium.

## Supplementary Materials

The following are available online at https://www.mdpi.com/article/10.3390/cancers13143579/s1, Figure S1: Cell death-EC50 of TQ in cell lines, Figure S2: Involvement of Bcl-2 in cell lines sensitivity to TQ, Figure S3: Loading control for Fluo-4 AM, Figure S4: Effect of TNF-α blockage of and pharmacological inhibition of ER stress, SOCE, calcium uniporter, autophagy, JNK, and cathepsin protease on TQ-induced cell death in Toledo cells, and Figure S5: Effect of TQ on mitochondrial calcium uptake.
