# Supplementary material for: Apoptotic and Non-Apoptotic Modalities of Thymoquinone-Induced Lymphoma Cell Death: Highlight of the Role of Cytosolic Calcium and Necroptosis
Source: Cancers (Basel). 2021 Jul 16;13(14):3579. doi: 10.3390/cancers13143579 (PMC8304872; doi:10.3390/cancers13143579)
Supplement: Supplementary file 1 [file cancers-13-03579-s001.zip › cancers-1242228_supplementary.pdf]

cancers
MDPI

Figure S1.

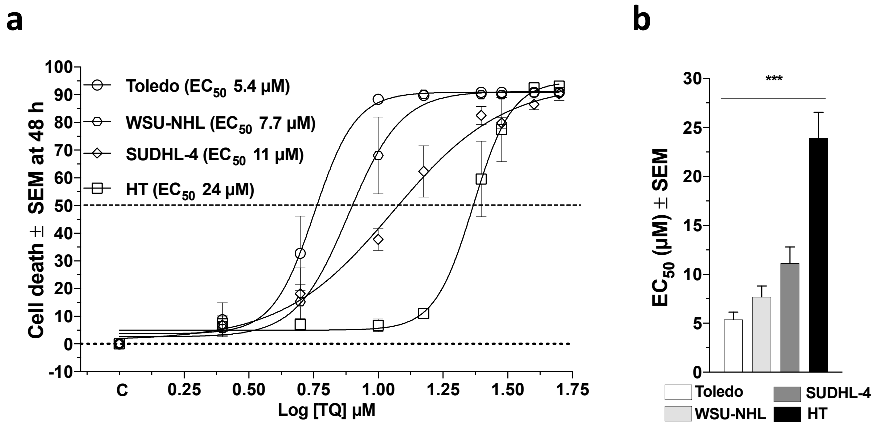

Figure S2.

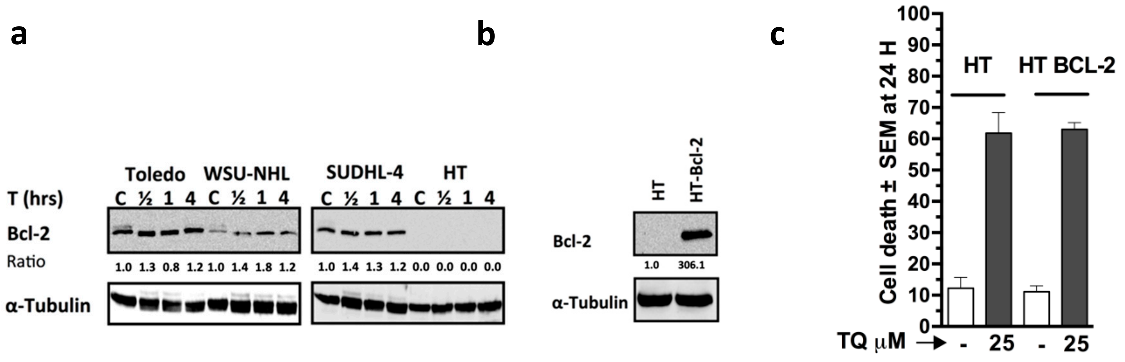

Figure S3.

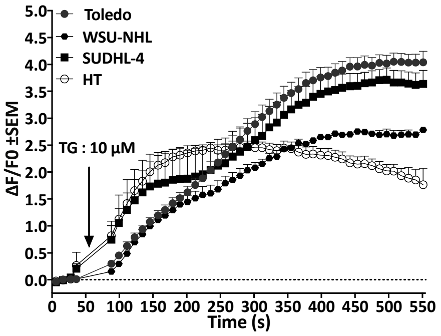

Figure S1. Cell death-EC_{50} of TQ in cell lines: cells were treated with increasing concentrations of TQ for 48 h, and cell death was monitored as in Figure 1a. **(a)** Dose-stimulation curves and the corresponding EC_{50} for each cell line. **(b)** Comparison of EC_{50} values between the different cell lines (n=3). ANOVA: *** $p < 0.001$.

Figure S2. Involvement of Bcl-2 in cell lines sensitivity to TQ: **(a)** Treated cell lines as indicated in Figure 2 were subjected to WB to detect Bcl-2. **(b)** WB analysis of Bcl-2 in HT and HT-Bcl-2 cells; HT-Bcl-2 cells were derived from HT cells after stable transfection with Bcl-2. **(c)** HT and HT-Bcl-2 cells were treated with 25 μM TQ for 24 h, and cell death was quantified as described above.

Figure S3. Loading control for Fluo-4 AM: To ensure correct Fluo-4 AM loading, cell lines were loaded with FLUO-4 AM and subjected to flow cytometry analysis in the presence of TG added at 60 sec; cell analysis and data processing were performed as indicated in Figure 4.

Figure S4.

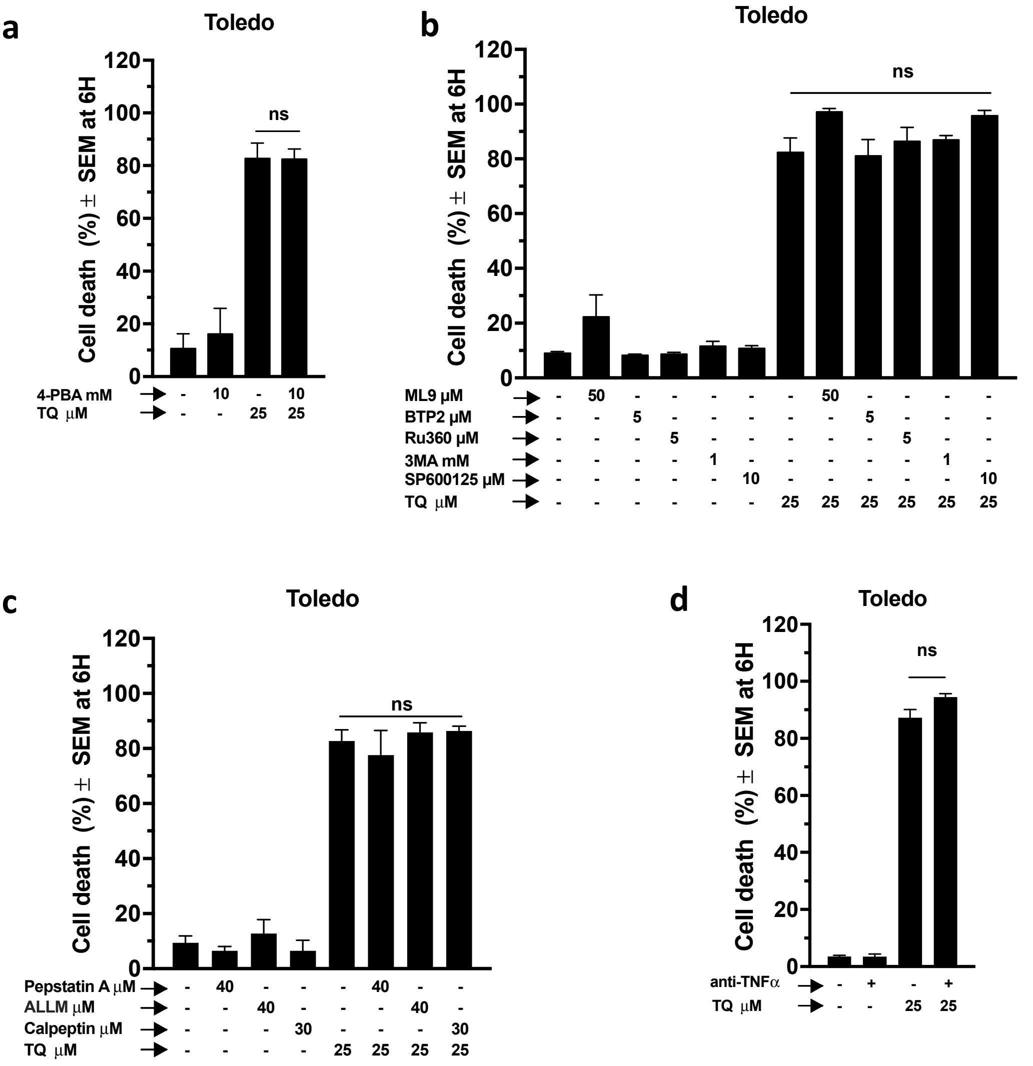

Figure S5.

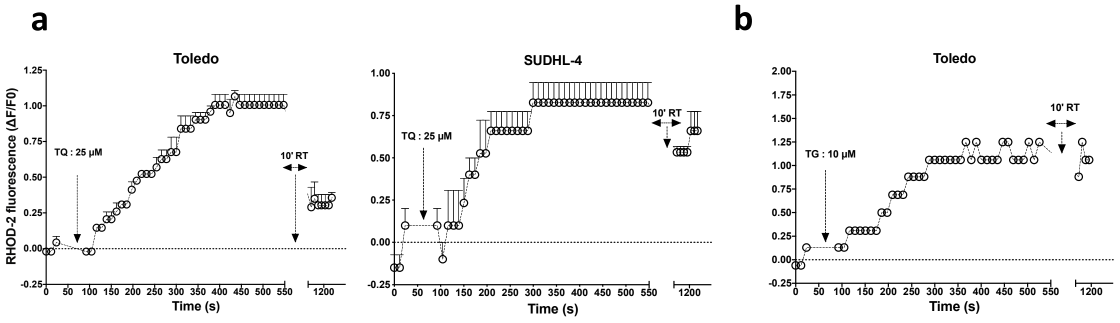

Figure S4. Effect of TNF-α blockage of and pharmacological inhibition of ER stress, SOCE, calcium uni-porter, autophagy, JNK, and cathepsin protease on TQ-induced cell death in Toledo cells**:** TQ-induced cell death was analyzed in Toledo cells pretreated with the ER stress inhibitor 4-PB shown in **(a)**, SOCE inhibitors (ML-9, BTP2), a calcium uniporter inhibitor (RU360), an autophagy inhibitor (3MA), a JNK inhibitor (SP600125) as shown in **(b)**, cathepsin proteases inhibitors as shown in **(c)** and with anti-TNF antibody at a concentration of 1 μg/mL as shown in **(d)**. Student's *t*-test: ns $p > 0.05$ in (a) and (d); ANOVA: ns $p > 0.05$ in (b) and (c).

Figure S5. Effect of TQ on mitochondrial calcium uptake. Toledo and SUDHL-4 cells were loaded with Rhod-2 and subjected to flow cytometry analysis in the presence of TQ added at 60 sec in **(a)**; in **(b)**, Toledo cells were examined in similar manner by TG. The means of 3 independent experiments are shown.

Table S1. List of genes (Human Apoptosis pathway 96 StellARrayTM qPCR Array list of genes).

**ID**	**Gene Name**
API5	API5-like 1; apoptosis inhibitor 5
BCL10	B-cell CLL/lymphoma 10; hypothetical LOC646626
BCL2	B-cell CLL/lymphoma 2
BCL6	B-cell CLL/lymphoma 6
BBC3	BCL2 binding component 3
BAK1	BCL2-antagonist/killer 1; BCL2-like 7 pseudogene 1
BAX	BCL2-associated X protein
BAD	BCL2-associated agonist of cell death
BAG1	BCL2-associated athanogene
BAG3	BCL2-associated athanogene 3
BAG4	BCL2-associated athanogene 4
BIK	BCL2-interacting killer (apoptosis-inducing)
BCL2L1	BCL2-like 1
BCL2L10	BCL2-like 10 (apoptosis facilitator)
BCL2L11	BCL2-like 11 (apoptosis facilitator)
BCL2L14	BCL2-like 14 (apoptosis facilitator)
BCL2L2	BCL2-like 2
BOK	BCL2-related ovarian killer
BCL2A1	BCL2-related protein A1
BNIP1	BCL2/adenovirus E1B 19kDa interacting protein 1
BNIP2	BCL2/adenovirus E1B 19kDa interacting protein 2
BNIP3	BCL2/adenovirus E1B 19kDa interacting protein 3
BNIP3L	BCL2/adenovirus E1B 19kDa interacting protein 3-like
BID	BH3 interacting domain death agonist
CD40	CD40 molecule, TNF receptor superfamily member 5
DPF2	D4, zinc and double PHD fingers family 2
DFFA	DNA fragmentation factor, 45kDa, alpha polypeptide
DDIT3	DNA-damage-inducible transcript 3
E2F1	E2F transcription factor 1
E2F2	E2F transcription factor 2
FAS	Fas (TNF receptor superfamily, member 6)
FADD	Fas (TNFRSF6)-associated via death domain
FASLG	Fas ligand (TNF superfamily, member 6)
HTRA2	HtrA serine peptidase 2
NAIP	NLR family, apoptosis inhibitory protein
PERP	PERP, TP53 apoptosis effector
PAWR	PRKC, apoptosis, WT1, regulator
RAD21	RAD21 homolog (S. pombe)
TRAF2	TNF receptor-associated factor 2

TRAF4	TNF receptor-associated factor 4
TRADD	TNFRSF1A-associated via death domain
XIAP	X-linked inhibitor of apoptosis
AVEN	apoptosis, caspase activation inhibitor
APAF1	apoptotic peptidase activating factor 1
BIRC2	baculoviral IAP repeat-containing 2
BIRC3	baculoviral IAP repeat-containing 3
BIRC5	baculoviral IAP repeat-containing 5
BFAR	bifunctional apoptosis regulator
CASP2	caspase 2, apoptosis-related cysteine peptidase
CASP3	caspase 3, apoptosis-related cysteine peptidase
CASP7	caspase 7, apoptosis-related cysteine peptidase
CASP8	caspase 8, apoptosis-related cysteine peptidase
CASP9	caspase 9, apoptosis-related cysteine peptidase
CARD10	caspase recruitment domain family, member 10
CLU	clusterin
DAP	death-associated protein
DAPK1	death-associated protein kinase 1
DAPK2	death-associated protein kinase 2
DAD1	defender against cell death 1
DIABLO	diablo homolog (Drosophila)
FGFR3	fibroblast growth factor receptor 3
FOXO3	forkhead box O3; forkhead box O3B pseudogene
GPX1	glutathione peroxidase 1
GSK3B	glycogen synthase kinase 3 beta
GADD45G	growth arrest and DNA-damage-inducible, gamma
HRK	harakiri, BCL2 interacting protein (contains only BH3 domain)
HSPA1A	heat shock 70kDa protein 1A; heat shock 70kDa protein 1B
HIPK2	homeodomain interacting protein kinase 2; similar to homeodomain interacting protein kinase 2
HIF1A	hypoxia inducible factor 1, alpha subunit (basic helix-loop-helix transcription factor)
IGF1	insulin-like growth factor 1 (somatomedin C)
LTA	lymphotoxin alpha (TNF superfamily, member 1)
LTBR	lymphotoxin beta receptor (TNFR superfamily, member 3)
MCL1	myeloid cell leukemia sequence 1 (BCL2-related)
NFKB1	nuclear factor of kappa light polypeptide gene enhancer in B-cells 1
PRDX2	peroxiredoxin 2
PMAIP1	phorbol-12-myristate-13-acetate-induced protein 1
PTEN	phosphatase and tensin homolog; phosphatase and tensin homolog pseudogene 1
RIPK1	receptor (TNFRSF)-interacting serine-threonine kinase 1
STAT5A	signal transducer and activator of transcription 5A
STAT5B	signal transducer and activator of transcription 5B

SIRT1	sirtuin (silent mating type information regulation 2 homolog) 1 (S. cerevisiae)
SOD1	superoxide dismutase 1, soluble
TGFB1	transforming growth factor, beta 1
TNF	tumor necrosis factor (TNF superfamily, member 2)
TNFRSF10A	tumor necrosis factor receptor superfamily, member 10a
TNFRSF11B	tumor necrosis factor receptor superfamily, member 11b
TNFRSF1A	tumor necrosis factor receptor superfamily, member 1A
TNFRSF1B	tumor necrosis factor receptor superfamily, member 1B
TNFAIP3	tumor necrosis factor, alpha-induced protein 3
TP53	tumor protein p53
TP53INP1	tumor protein p53 inducible nuclear protein 1
UNC5B	unc-5 homolog B (C. elegans)
AKT1	v-akt murine thymoma viral oncogene homolog 1
VEGFA	vascular endothelial growth factor A
